# Gridded birth and pregnancy datasets for Africa, Latin America and the Caribbean

**DOI:** 10.1038/sdata.2018.90

**Published:** 2018-05-22

**Authors:** W.H.M. James, N. Tejedor-Garavito, S.E. Hanspal, A. Campbell-Sutton, G.M. Hornby, C. Pezzulo, K. Nilsen, A. Sorichetta, C.W. Ruktanonchai, A. Carioli, D. Kerr, Z. Matthews, A.J. Tatem

**Affiliations:** 1 WorldPop, Geography and Environment, University of Southampton, Highfield Campus, Southampton SO17 1BJ, UK; 2 Flowminder Foundation, Roslagsgatan 17, Stockholm SE-11355, Sweden; 3 GeoData, University of Southampton, Highfield Campus, Southampton SO17 1BJ, UK; 4 Division of Social Statistics and Demography & Centre for Global Health, Population, Poverty and Policy, Faculty of Social and Human Sciences, University of Southampton, Southampton SO17 1BJ, UK

**Keywords:** Developing world, Geography, Databases

## Abstract

Understanding the fine scale spatial distribution of births and pregnancies is crucial for informing planning decisions related to public health. This is especially important in lower income countries where infectious disease is a major concern for pregnant women and new-borns, as highlighted by the recent Zika virus epidemic. Despite this, the spatial detail of basic data on the numbers and distribution of births and pregnancies is often of a coarse resolution and difficult to obtain, with no co-ordination between countries and organisations to create one consistent set of subnational estimates. To begin to address this issue, under the framework of the WorldPop program, an open access archive of high resolution gridded birth and pregnancy distribution datasets for all African, Latin America and Caribbean countries has been created. Datasets were produced using the most recent and finest level census and official population estimate data available and are at a resolution of 30 arc seconds (approximately 1 km at the equator). All products are available through WorldPop.

## Background & Summary

Accurate and detailed information on the spatial distribution and numbers of births and pregnancies is crucial for informing planning decisions related to public health^1^. The survival and health of women and their new-born babies in low income countries is a key priority, with the reduction of maternal and neonatal mortality central for meeting a number of the United Nations Sustainable Development Goals (specifically goals 3.1 and 3.2)^2^. Whilst progress has been made, there were still 303,000 maternal deaths in 2015 (ref. 3) and children in lower income countries are 14 times more likely to die during their first 28 days of life compared to their higher income counterparts. Despite this, the spatial detail of basic data on the numbers and distribution of births and pregnancies is often of a coarse resolution and difficult to obtain^4^, with no co-ordination between countries and organisations to create one consistent set of subnational estimates for planning.

Whilst there are clear inequalities of maternal and neonatal healthcare between nations^5^, there are also large disparities within individual countries, with growing recognition that national levels and trends could be masking important sub-national variations^6^. For example, a study in Indonesia found that under-5 mortality was nearly four times higher in the poorest fifth of the population than in the richest fifth^7^, and gaps like these are more likely to occur at the sub-national level^7–9^. Although progress has been made in reducing such inequalities, there is still substantial work to be done. As such, understanding sub-national variation and inequity in health status, wealth and access to resources is increasingly being recognised as central to meeting developmental goals^10^. To understand and tackle inequalities related to maternal and neonatal health, the first step is to have a detailed knowledge of the distribution of births and pregnancies, which is known to vary substantially due to population age and sex distribution and age specific fertility rates (ASFR)^4^. These are also valuable data for subnational planning and estimation, and calculation of subnational indicators that rely on births or pregnancies as a denominator. When considering maternal and neonatal health in lower income countries, infectious disease is a major concern as pregnant women and new-borns are particularly at risk from many diseases, such as malaria^11^ and HIV^12^. This issue has recently been highlighted by the Zika virus outbreak in Latin America, further intensifying the need for detailed information on the number and distribution of births and pregnancies. Currently there is a clear lack of data for such analysis, with complete and continuous datasets of numbers of births only available at the national level (e.g., United Nations Population Division^13^). Whilst sub-national datasets are readily available for some countries, their spatial detail is often coarse with differences in the recorded metrics, sampling framework and data formats meaning that it is extremely difficult to assess burden within and across multiple nations.

This study aims to overcome the data gap identified above by producing continental scale, gridded datasets of numbers of births and pregnancies with a spatial resolution of 30 arc seconds (approximately 1 km at the equator). Advances in computational power and spatial econometric techniques, as well as the increasing availability of geo-located data, have increased the ability to produce these fine spatial resolution datasets. As such, in the framework of the WorldPop project (www.worldpop.org), and extending the approaches described by Tatem *et al.*^4^, an open access archive of gridded birth and pregnancy distribution datasets for all African, Latin America and Caribbean (LAC) countries has been created. This process used the most recent and finest level census, census microdata, household survey data and official population estimate data available to the authors at the time of writing, alongside a range of geospatial datasets.

## Methods

**Figure 1:**
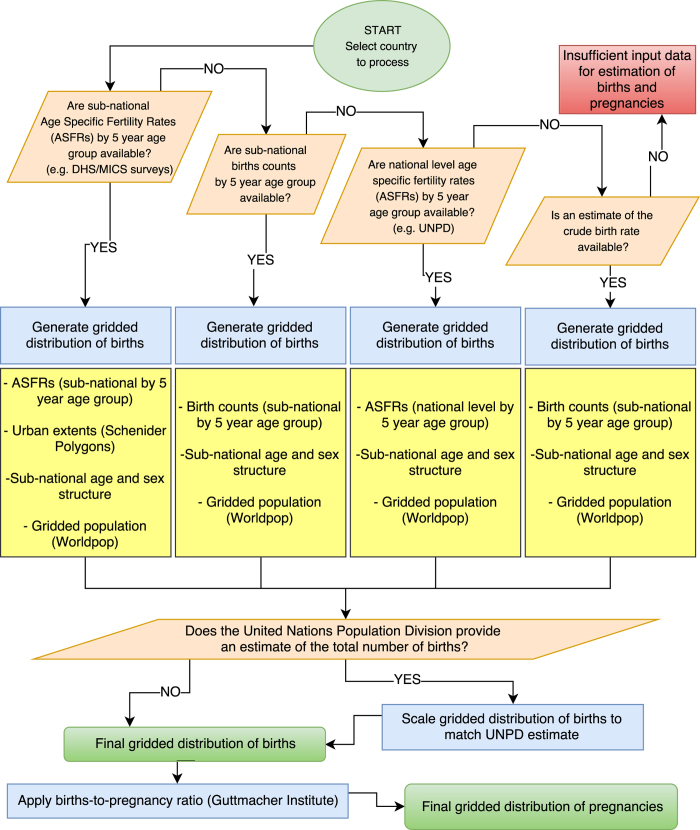
Schematic overview of the workflow adopted to generate gridded subnational births and pregnancy datasets. ASFR=Age Specific Fertility Rate, DHS=Demographic and Health Survey, MICS=Multiple Indicator Cluster Survey, UNPD=United Nations Population Division.

Gridded estimates of live births were produced for 50 Latin American and Caribbean and 58 African countries at a spatial resolution of 30 arc seconds. This was achieved by combining the latest datasets on population distribution, population age and sex structure and fertility rates in a GIS environment. Estimates of pregnancies were additionally generated using national-level estimates for stillbirths, miscarriages and abortions from the Guttmacher Institute^14^. The workflow develops the methods presented by Tatem *et al.*^4^, using a variety of data sources to construct continent wide datasets. The process is fully automated by a Python Script, allowing the rapid processing of multiple countries and alignment to a standard grid for the production of seamless continental scale datasets. The workflow is shown in Fig. 1 and described in detail below. Maps of the data sources and date for each country and whether urban and rural ASFR estimates were available can be found in the Supplementary Figures 1 and 2 respectively.

### The basis for estimation: population distributions

**Figure 2:**
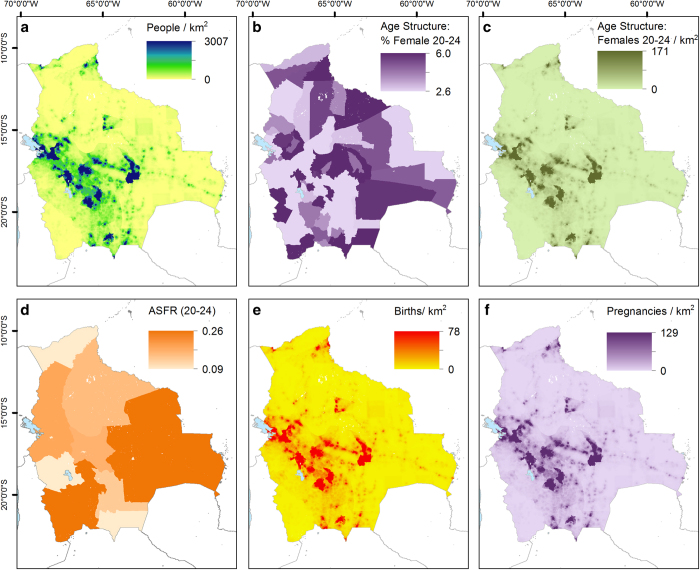
Examples of input, intermediate and output datasets for Bolivia. (**a**) Gridded population input (WorldPop), (**b**) Age and sex structure disaggregated by administrative unit, (**c**) Derived gridded age and sex structure, (**d**) ASFRs disaggregated by region and urban vs rural, (**e**) Gridded births output, (**f**) Gridded pregnancies output.

The population distribution forms a major component of the births and pregnancy estimation process. The WorldPop project has recently completed construction of gridded population distribution datasets for all low- and middle-income countries at a resolution of 30 arc seconds. Full details are provided on the WorldPop website (www.worldpop.org.uk) along with links describing the methods in detail^15–17^. This study uses the relevant regions of Africa (Data Citation 1) and Latin America and the Caribbean (Data Citation 2), whose total population is adjusted to match the most recent United Nations Population Division (UNPD)^18^ 2015 estimates available when the population distribution datasets were produced. Figure 2a shows the gridded population distribution dataset for Bolivia as an example. To ensure data consistency, a WorldPop standard grid was used in processing; this is a gridded dataset providing ISO country codes at a resolution of 30 arc second (Data citation 3).

### Calculating the proportion of women of reproductive age

Sub-national information on age and sex structure was collected, specifically women of childbearing age grouped in seven 5-years age groups (i.e., 15–19, 20–24, 25–29, 30–34, 35–39, 40–44, 45–49), as defined by the UNPD^18^. Datasets for the majority of Africa were provided by Pezzulo *et al.*^19^ whilst datasets for the remaining African, Latin America and the Caribbean countries were assembled from a variety of sources, following the protocols defined by Pezzulo *et al.*^19^. Table 1 (available online only) shows the source, spatial detail (i.e., administrative unit level) and reference year used for the countries processed in this study.

With the raw data recorded and documented according to different protocols determined by national governments, the project was presented with a wide range of table data formats and schemas. Data restructuring was achieved using scripting (R 3.3.1, Python 2.7) and table processing software (Microsoft Excel 2013). The resultant standardised tables contained fields corresponding to the proportionate values of people (both sexes) in each 5-year age group, and the overall proportion of males and females in each region. Table 2 shows an example of the standardised tables, for regions of Peru.

Age and sex structure information was matched to vector geographical boundaries from the Global Administrative Areas (GADM) database^20^, with the exceptions of Chile and Colombia where boundaries from the National Statistics Office were used. The extent of these boundaries was standardised to those defined by the WorldPop gridded ISO country code dataset using the Clip and Nibble tools in ArcGIS 10.3, executed as part of the Python^21^ script. Figure 2b shows the distribution of females between the ages of 20 and 24 for Bolivia. Similar distributions were created for all other 5-year age groupings in the 15–49-year range.

### Estimating fertility rates

Data on fertility was collected on a country-by-country basis to provide the most up to date and spatially detailed information. Data sources were chosen using a hierarchical approach as shown in Fig. 1, prioritising sources which included information on age specific fertility and those of the highest spatial detail. The type of fertility data used for each country is shown in Table 3 (available online only) and described in detail below. As with the age and sex structure datasets, restructuring and table processing was carried out using a variety of scripting and software packages (Python 2.7, R 3.3.1, Microsoft Excel 2013) to produce a common format and schema for each data type, as described in detail below.

To provide the finest spatial detail of the distribution of fertility across each country, ASFRs were estimated by 5-year age groups, disaggregated sub-nationally according to the relevant Demographic and Health Surveys (DHS), Multiple Indicator Cluster Surveys (MICS) or National Statistics Agency survey regions and by urban vs rural if available. Table 3 (available online only) indicates which countries had the required data for estimation. ASFRs for each 5-year age group were commonly derived from DHS or MICS, with the exceptions of Aruba, Chile and Eritrea where the relevant datasets were available from the corresponding National Statistics Office.

For DHS and MICS surveys, ASFRs were estimated using a Stata program developed by Pullum^50^, as discussed in Tatem *et al.*^4^. The program calculated the basic demographic indicator by deriving ASFRs for each of the seven 5-year age groups covering the reproductive life span from 15–49 years based on dividing the number of births to women in each age group, during a retrospective 3-years reference period, by the number of women-years during the same period. Data restructuring and table processing was carried out using R 3.3.1 and Microsoft Excel 2013 to produce a common format, as that shown in Table 4.

Datasets representing the boundaries of subnational regions (Table 3) (available online only) were assembled and the relevant ASFRs matched to them. If the ASFR data was available for urban and rural areas within the subnational regions, the MODIS 500 m Global Urban Extent dataset^51^ was used to distinguish urban and rural areas and allocate the constant value within them. Figure 2d shows an example ASFR dataset for Bolivia, showing the ASFRs for one reproductive age range: the 20–24 age group by sub-region. Similar datasets were constructed for all other 5-year age groups within the 15 to 49 range.

For countries where ASFRs disaggregated sub-nationally and by urban/rural were not available, information on the spatial variation of age structured fertility was sought from vital registration systems, census records and other national sources. This was routinely in the form of births registered per administrative unit per 5-year age grouping. Table 3 (available online only) shows for which countries this type of data (registered births per age group) was used, with Table 5 showing an example of the standardised table format for Venezuela.

Sub-national ASFRs were calculated by dividing the number of births in each age group (e.g., Table 5) by the number of females in the corresponding group. The latter was derived from the WorldPop population distribution^15^ and age-sex distributions produced following the methodology of Pezzulo *et al.*^19^ and outlined in Table 1 (available online only).

The UNPD provides national estimates of AFSRs by 5-year age grouping for the majority of countries^18,24^. These datasets were used where subnational information was not available. As with all other datasets, the country boundaries defined by WorldPop^15^ were used to define the geographical extent.

For 9 countries, there was no information available on age specific fertility, either sub-nationally or nationally. In these cases, crude birth rates were obtained from a variety of official sources (Table 3 (available online only)) which were subsequently matched to the appropriate GIS country boundaries supplied by WorldPop^15^.

### Estimating the number of births from fertility, population and age structure

For countries where measures of age specific fertility were available, the distribution of live births was estimated by multiplying the number of females in each age group (e.g., Fig. 2c) by the corresponding ASFR gridded dataset (e.g., Fig. 2d) or value (in the case of national ASFR estimates). The resultant seven age specific gridded datasets were summed to generate an estimate of total births. For countries where fertility was expressed simply as a crude birth rate (Table 3 (available online only)), the births distribution was calculated by multiplying the crude birth rate by the initial 30 arc second UNPD adjusted WorldPop population grid for 2015 (ref. 15).

**Figure 3:**
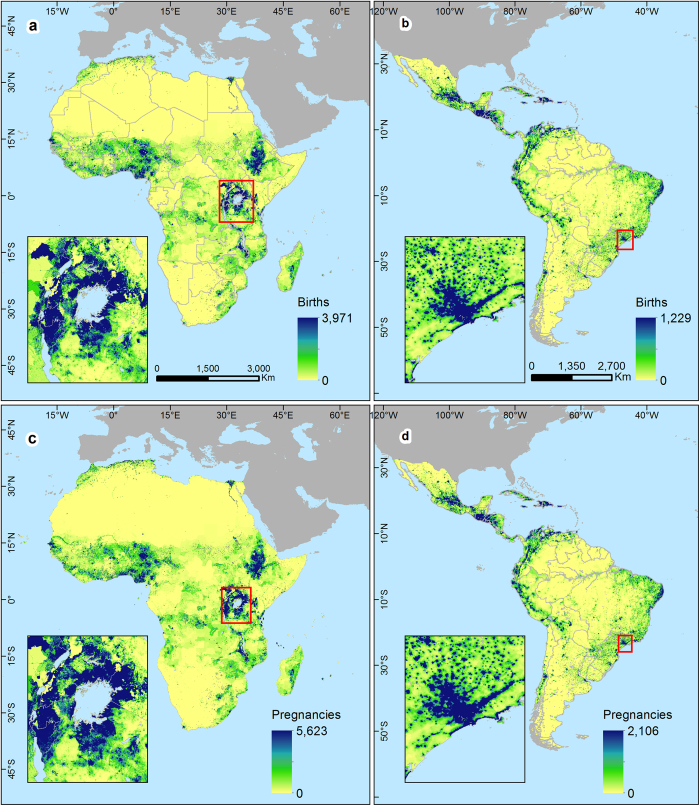
Estimated births and pregnancies per grid cell for Africa, Latin America and the Caribbean in 2015. The grid cell resolution is 30 arc seconds (approximately 1 km at the equator) and co-ordinates refer to GCS WGS 1984.

Finally, for each country, the distribution was scaled to match the UNPD estimate of the total number of births^18^. For countries where the UNPD does not provide an estimate of the total number of births, the initial total was used. An example of the final distributed births gridded dataset for Bolivia is shown in Fig. 2e, with results for Africa, Latin America and the Caribbean shown in Fig. 3 and Supplementary Table 1.

### Estimating the distribution of pregnancies

The Guttmacher institute has published country specific estimates of the number of stillbirths, miscarriages and abortions at the national level^14^. These estimates for 2014 were integrated with UNPD national estimates on numbers of live births^18^ to construct a ratio between numbers of births and pregnancies. This ratio was applied to the live births distribution to generate an estimate of the distribution of pregnancies. For countries not covered by the Guttmacher dataset, the nearest suitable geographical country value was used. An example of the final distributed pregnancies gridded dataset for Bolivia is shown in Fig. 2f whilst results for the entire Africa, Latin America and the Caribbean are shown in Fig. 3.

### Code availability

The Python code developed for production of the births and pregnancies datasets is publicly and freely available through Figshare^52^. The code consists of a Python programming language script (version 2.7; www.python.org) and relies on the ArcGIS 10.4.1 ArcPy site package for performing GIS specific spatial operations. The script is internally documented to both explain its purpose (including a description of the GIS-specific spatial operations it performs) and, when required, guiding the user through its customisation.

## Data Records

The high-resolution births and pregnancies datasets described in this article referring to the 108 countries listed in Table 3 (available online only) are publicly and freely available through the WorldPop Repository (http://www.worldpop.org.uk/data/). A collection of these datasets has been compiled for the births for LAC (Data Citation 4) and Africa (Data Citation 5) and pregnancies for LAC (Data Citation 6) and Africa (Data Citation 7), as described in Table 6.

## Technical Validation

All data collected, assembled and used were (i) already validated by the corresponding data collector, owner and/or distributor, and (ii) further checked, in the framework of this project. The gridded 5-year age and sex count datasets constructed for Latin America and the Caribbean (e.g., Fig. 2c) were verified following the protocol outlined in Pezzulo *et al.*^19^, who compiled and assessed similar datasets for Africa and Asia. Briefly, this comprised of summing all the layers into a single dataset (representing the total numbers of people for all age and sex groups at the grid cell level) and then subtracting it from the corresponding WorldPop continental gridded population count dataset to make sure that the country totals matched the UNPD estimates for the year in question. All fertility rates used in this study were checked, on a county-by-country basis, to make sure they were within reasonable ranges. Additionally, for countries where additional sources of fertility data were available, estimates were produced using all available sources to compare the adjusted total births. These results showed that, although differences may be observed at the grid cell level, the totals at the administrative unit level are very similar. Endeavours were made to assemble the most recent, reliable and spatially detailed data at the time of writing. However, additional input from readers who may have knowledge or access to more recent and/or better datasets are welcome for improving future iterations of the outputs.

The accuracy and quality of fertility estimates from survey data such as those provided by the DHS, have been assessed in several reports, by testing the quality of the birth history data in a large number of countries. These checks were mainly aimed to identify potential omission and displacement of births, potential displacement of births, or misreporting of date of birth^53,54^. Overall, although a number of issues were identified for some countries, these studies found that most estimates were either good or of acceptable quality. Furthermore, outcomes from Pullum and Becker^53^ show that in general the latest DHS surveys are less prone to issues like incomplete birthdates, omissions and displacement of births and deaths. Similarly, a more recent report from Pullum and Staveteig^55^, exploring the quality and consistency of age and date reports in DHS surveys, demonstrates that DHS data is constantly evaluated to improve its quality.

**Figure 4:**
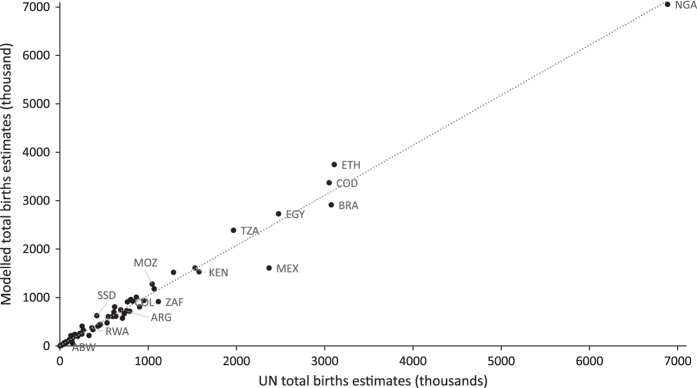
Total number of births per country estimated by this study plotted against the corresponding UNPD estimate.

Modelled estimates of total number of births per country prior to adjustments (to match UNPD estimate) were also plotted against the UNPD estimates^18^ to assess the size of differences obtained through using subnational data sources. Figure 4 shows the correlation for the 95 countries for which UNPD provides an estimate, with a corresponding R^2^ value of 0.982. Analysis was not possible for the 9 countries for which the UNPD does not provide an estimate, although these make up a very small proportion (0.01%) of the total births across the whole study area.

## Usage Notes

The datasets presented here can be used both to (i) support applications measuring sub-national metrics of maternal and new-born health and (ii) to inform planning decisions. However, considering that they represent modelling outputs generated using ancillary covariates for producing the underlying WorldPop population distribution datasets, to avoid circularity, they should not be used to make predictions or explore relationships about any of those ancillary datasets^56^. Thus, before using the births and pregnancies datasets in correlation analyses against factors which are included in the construction of the population distribution datasets (e.g., correlating birth distribution with land-cover), ideally the population modelling process should be re-run using the WorldPop-RF code^57^ with the applicable covariates removed.

## Additional information

**How to cite this article**: James, W. H. M. *et al.* Gridded birth and pregnancy datasets for Africa, Latin America and the Caribbean. *Sci. Data* 5:180090 doi: 10.1038/sdata.2018.90 (2018).

**Publisher’s note**: Springer Nature remains neutral with regard to jurisdictional claims in published maps and institutional affiliations.

## Supplementary Material



Supplementary Information

## Figures and Tables

**Table 1:** Data sources for African, Latin American and Caribbean (LAC) countries from which age and sex proportions were derived

**Country**	**ISO Code**	**Continent**	**Data Type Used**	**Year**	**Administrative Unit Level (number of units)**	**Data Source**
Anguilla	AIA	LAC	Census	2001	0 (1)	Statistics Department, Government of Anguilla
Antigua and Barbuda	ATG	LAC	Census	2011	0 (1)	Statistics Division, Government of Antigua and Barbuda
Argentina	ARG	LAC	Census	2010	2 (528)	Instituto Nacional de Estadística y Censos, Argentina
Aruba	ABW	LAC	Census	2010	0 (1)	Central Bureau of Statistics, Aruba
Bahamas	BHS	LAC	Census	2010	1 (32)	Department of Statistics, The Commonwealth of The Bahamas
Barbados	BRB	LAC	Census	2010	1 (11)	Barbados Statistical Service
Belize	BLZ	LAC	Census	2010	1 (6)	Statistical Institute of Belize
Bolivia	BOL	LAC	Census	2012	2 (95)	Instituto Nacional de Estadística, Bolivia
Bonaire, Saint Eustatius and Saba	BES	LAC	Annual Stats	2016	1 (3)	Central Bureau of Statistics: Netherlands Antilles and Island Registries
Brazil	BRA	LAC	Census	2010	2 (5570)	Instituto Brasileiro de Geografia e Estatística
British Virgin Islands	VGB	LAC	Census	2010	0 (1)	Central Statistics Office, Government of the Virgin Islands
Cayman Islands	CYM	LAC	Census	2010	0 (1)	Economics and Statistics Office, Cayman Islands Government
Chile	CHL	LAC	Census	2014	1 (16)	Instituto Nacional de Estadísticas, Chile
Colombia	COL	LAC	Census	2005	1 (33)	National Administrative Department of Statistics, Colombia
Costa Rica	CRI	LAC	Census	2011	1 (7)	Instituto Nacional de Estadística y Censos, Costa Rica
Cuba	CUB	LAC	Census	2012	1 (16)	La Oficina Nacional de Estadísticas de Cuba
Curacao	CUW	LAC	Census	2011	0 (1)	Central Bureau of Statistics, Curaçao
Dominica	DMA	LAC	Census	2011	1 (10)	Central Statistics Office, Dominica
Dominican Republic	DOM	LAC	Census	2010	2 (155)	Oficina Nacional de Estadística, Dominican Republic
Ecuador	ECU	LAC	Census	2010	3 (1024)	Instituto Nacional de Estadísticas, Ecuador
El Salvador	SLV	LAC	Annual Stats	2009	1 (14)	Directorate-General for Statistics and Census, El Salvador
Falkland Islands	FLK	LAC	Census	2012	0 (1)	Falkland Islands Government
French Guiana	GUF	LAC	Annual Stats	2016	0 (1)	Institut National de la Statistique et des Etudes Economiques, France
Grenada	GRD	LAC	Census	2001	1 (7)	Regional Statistics Sub-Programme, Caribbean Community
Guadeloupe	GLP	LAC	Annual Stats	2016	0 (1)	Institut National de la Statistique et des Etudes Economiques, France
Guatemala	GTM	LAC	Census	2002	2 (352)	Instituto Nacional de Estadística, Guatemala
Guyana	GUY	LAC	Census	2012	1 (10)	Bureau of Statistics, Guyana
Haiti	HTI	LAC	Census	2001	1 (10)	Institut Haïtien de Statistique et d'Informatique, Haïti
Honduras	HND	LAC	Census	2013	1 (18)	Instituto Nacional de Estadística, Honduras
Jamaica	JAM	LAC	Census	2011	1 (14)	Statistical Institute of Jamaica
Martinique	MTQ	LAC	Annual Stats	2016	0 (1)	Institut National de la Statistique et des Etudes Economiques, France
Mexico	MEX	LAC	Census	2010	1 (32)	Instituto Nacional de Estadística y Geografía, México
Montserrat	MSR	LAC	Census	2011	0 (1)	Statistics Department, Montserrat
Nicaragua	NIC	LAC	Census	2005	2 (137)	Instituto Nacional de Información de Desarrollo, Nicaragua
Panama	PAN	LAC	Census	2010	1 (13)	Instituto Nacional de Estadística y Censos, Panamá
Paraguay	PRY	LAC	Census microdata	2002	1 (18)	Integrated Public Use Microdata Series, International (IPUMSI)
Peru	PER	LAC	Census	2007	2 (194)	Instituto Nacional de Estadística e Informática, Peru
Puerto Rico	PRI	LAC	Census	2010	1 (78)	Instituto de Estadísticas de Puerto Rico
Saint Barthelemy	BLM	LAC	Annual Stats	2013	0 (1)	Institut National de la Statistique et des Etudes Economiques, France
Saint Kitts and Nevis	KNA	LAC	Census	2001	1 (14)	Regional Statistics Sub-Programme, Caribbean Community
Saint Lucia	LCA	LAC	Census	2010	0 (1)	Central Statistics Office, Saint Lucia
Saint Martin	MAF	LAC	Census	2013	0 (1)	Institut National de la Statistique et des Etudes Economiques, France
Saint Vincent and The Grenadines	VCT	LAC	Census	2012	1 (6)	Statistics Office, St. Vincent
Sint Maarten	SXM	LAC	Census	2011	0 (1)	Department of Statistics, Sint Maarten
Suriname	SUR	LAC	Census	2012	2 (62)	General Bureau of Statistics, Suriname
Trinidad and Tobago	TTO	LAC	Census	2000	1 (15)	Central Statistical Office, Trinidad and Tobago
Turks and Caicos Islands	TCA	LAC	Census	2012	0 (1)	Statistical Office, Turks and Caicos Islands
Uruguay	URY	LAC	Census	2011	2 (231)	Instituto Nacional de Estadística, Uruguay
Venezuela	VEN	LAC	Census	2011	2 (337)	Instituto Nacional de Estadística, Venezuela
Virgin Islands, U.S.	VIR	LAC	Census	2010	2 (20)	U.S. Census Data and Statistics, USA
Algeria*	DZA	Africa	Census	2008	1 (48)	Office National des Statistique, Algeria
Angola*	AGO	Africa	Demographic and Health Surveys- Malaria Indicators Surveys	2011	1 (18)	MEASURE Demographic and Health Surveys, USAID
Benin*	BEN	Africa	Demographic and Health Surveys	2011	1 (12)	MEASURE Demographic and Health Surveys, USAID
Botswana*	BWA	Africa	Census	2006	2 (21)	Central Statistics Office, Botswana
Burkina Faso*	BFA	Africa	Census	2006	1 (13)	Institut National de la Statistique et de la Demographie (INSD), Burkina Faso
Burundi*	BDI	Africa	Demographic and Health Surveys	2010	1 (17)	MEASURE Demographic and Health Surveys, USAID
Cameroon*	CMR	Africa	Demographic and Health Surveys	2011	1 (12)	MEASURE Demographic and Health Surveys, USAID
Cape Verde	CPV	Africa	Census	2010	1 (22)	Instituto Nacional de Estatística, Cape Verde
Central African Republic*	CAF	Africa	Multiple Indicator Cluster Surveys	2006	1 (17)	UNICEF
Chad*	TCD	Africa	Demographic and Health Surveys	2004	1 (8)	MEASURE Demographic and Health Surveys, USAID
Comoros	COM	Africa	Census	2001	0 (1)	Department of Statistics, Comoros
Congo*	COG	Africa	Demographic and Health Surveys-Aids Indicator Surveys	2009	1 (4)	MEASURE Demographic and Health Surveys, USAID
Congo, The Democratic Republic Of The*	COD	Africa	Demographic and Health Surveys	2013	1(11)	MEASURE Demographic and Health Surveys, USAID
Cote D'ivoire*	CIV	Africa	Demographic and Health Surveys-Aids Indicator Surveys	2005	1 (11)	MEASURE Demographic and Health Surveys, USAID
Djibouti*	DJI	Africa	Multiple Indicator Cluster Surveys	2006	1 (2)	UNICEF
Egypt*	EGY	Africa	Census microdata	2006	1 (26)	Integrated Public Use Microdata Series, International (IPUMSI)
Equatorial Guinea*	GNQ	Africa	United Nations	2010	0(1)	World Population Prospects, United Nations Population Division
Eritrea*	ERI	Africa	United Nations	2010	0 (1)	World Population Prospects, United Nations Population Division
Ethiopia*	ETH	Africa	Census	2007	1 (11)	Central Statistical Agency of Ethiopia
Gabon*	GAB	Africa	Demographic and Health Surveys	2012	1 (10)	MEASURE Demographic and Health Surveys, USAID
Gambia*	GMB	Africa	Multiple Indicator Cluster Surveys	2006	1 (13)	MEASURE Demographic and Health Surveys, USAID
Ghana*	GHA	Africa	Census—USCB	2010	2 (110)	United States Census Bureau, USAID
Guinea*	GIN	Africa	Demographic and Health Surveys	2012	1 (8)	MEASURE Demographic and Health Surveys, USAID
Guinea-Bissau*	GNB	Africa	Multiple Indicator Cluster Surveys	2006	1 (9)	UNICEF
Kenya*	KEN	Africa	Census—USCB	2010	1 (47)	United States Census Bureau, USAID
Lesotho*	LSO	Africa	Census	2004	1 (10)	Lesotho Bureau of Statistics
Liberia*	LBR	Africa	Census	2008	1 (15)	Liberia Institute of Statistics and Geo-Information Service
Libyan Arab Jamahiriya*	LBY	Africa	United Nations	2010	0 (1)	World Population Prospects, United Nations Population Division
Madagascar*	MDG	Africa	Demographic and Health Surveys	2009	1 (22)	MEASURE Demographic and Health Surveys, USAID
Malawi*	MWI	Africa	Census	2008	2 (350)	National Statistical Office, Malawi
Mali*	MLI	Africa	Demographic and Health Surveys	2012	1 (9)	MEASURE Demographic and Health Surveys, USAID
Mauritania*	MRT	Africa	Multiple Indicator Cluster Surveys	2007	1 (13)	UNICEF
Mauritius	MUS	Africa	Census	2011	1 (12)	National Statistics Office, Mauritius
Mayotte	MYT	Africa	Census	2016	0 (1)	Institut National de la Statistique et des Etudes Economiques, France
Morocco*	MAR	Africa	Census	2004	2 (15)	Haut Commissariat au Plan, Morocco
Mozambique*	MOZ	Africa	Census	2007	2 (129)	Instituto Nacional de Estatística, Mozambique
Namibia*	NAM	Africa	Census—USCB	2010	2 (102)	United States Census Bureau, USAID
Niger*	NER	Africa	Demographic and Health Surveys	2012	1 (8)	MEASURE Demographic and Health Surveys, USAID
Nigeria*	NGA	Africa	Census	2006	1 (37)	National Bureau of Statistics, Nigeria
Reunion	REU	Africa	Census	2016	0 (1)	Institut National de la Statistique et des Etudes Economiques, France
Rwanda*	RWA	Africa	Census—USCB	2010	2 (30)	United States Census Bureau, USAID
Saint Helena	SHN	Africa	Census	2008	2 (10)	National Statistics Office, Saint Helena
Sao Tome and Principe	STP	Africa	Census	2016	2 (7)	Instituto Nacional de Estatística (INE), São Tomé e Príncipe
Senegal*	SEN	Africa	Census microdata	2002	2 (30)	Integrated Public Use Microdata Series, International (IPUMSI)
Seychelles	SYC	Africa	Census	2016	0 (1)	National Bureau of Statistics, Seychelles
Sierra Leone*	SLE	Africa	Census	2004	2 (14)	Statistics Sierra Leone
Somalia*	SOM	Africa	Multiple Indicator Cluster Surveys	2006	1 (18)	UNICEF
South Africa*	ZAF	Africa	Census—USCB	2010	3 (259)	United States Census Bureau, USAID
South Sudan*	SSD	Africa	Census microdata	2008	1 (10)	Integrated Public Use Microdata Series, International (IPUMSI)
Sudan*	SDN	Africa	Census microdata	2008	1 (15)	Integrated Public Use Microdata Series, International (IPUMSI)
Swaziland*	SWZ	Africa	Demographic and Health Surveys	2007	1 (4)	MEASURE Demographic and Health Surveys, USAID
Tanzania, United Republic Of*	TZA	Africa	Census—USCB	2010	2(117)	United States Census Bureau, USAID
Togo*	TGO	Africa	Demographic and Health Surveys	2013	1 (6)	National Statistics Institute, Tunisia
Tunisia*	TUN	Africa	Census	2004	1 (24)	MEASURE Demographic and Health Surveys, USAID
Uganda*	UGA	Africa	Demographic and Health Surveys	2011	1 (10)	United States Census Bureau, USAID
Western Sahara*	ESH	Africa	United Nations	2010	0 (1)	World Population Prospects, United Nations Population Division
Zambia*	ZMB	Africa	Census—USCB	2010	3 (150)	United States Census Bureau, USAID
Zimbabwe*	ZWE	Africa	Demographic and Health Surveys	2011	1 (10)	MEASURE Demographic and Health Surveys, USAID
Note: Data marked with (*) are from Pezzulo et al.^19^						

**Table 2:** Example of standardised table containing proportionate values of people by age group and sex for Peru.

**Region**	**t_0_4**	**t_5_9**	**t_10_14**	**t_15_19**	**……**	**t_60_64**	**t_65_plus**	**prop_m_t**	**prop_f_t**
PER_Callao_Callao	0.09	0.08	0.09	0.09		0.03	0.06	0.49	0.51
PER_Cusco_Acomayo	0.12	0.14	0.14	0.08		0.03	0.08	0.49	0.51
PER_Cusco_Anta	0.10	0.12	0.14	0.10		0.03	0.08	0.50	0.50
PER_Cusco_Calca	0.11	0.12	0.14	0.10		0.02	0.06	0.50	0.50
PER_Cusco_Canas	0.12	0.14	0.14	0.09		0.03	0.08	0.50	0.50
PER_Cusco_Canchis	0.10	0.12	0.13	0.10		0.03	0.07	0.49	0.51
(Note table is for illustrative purposes only and therefore does not display all age groups).									

**Table 3:** Fertility data sources for all African, Latin American and Caribbean countries

**Country**	**ISO**	**Type**	**Measure**	**Source**	**Year**	**Number of Units**
Aruba	ABW	Census	ASFR	National institution^22^	2000	1
Angola	AGO	Survey sample	ASFR	DHS^23^	2011	8
Anguilla	AIA	Vital registration	ASFR	UNPD 2008^24^	2006	1
Argentina	ARG	Vital registration	Birth count	National institution^25^	2012	24
Antigua and Barbuda	ATG	National estimate	ASFR	UNPD 2015^18^	2010–15	1
Burundi	BDI	Survey sample	ASFR	DHS^23^	2012	9
Benin	BEN	Survey sample	ASFR	DHS^23^	2012	23
Bonaire, Saint Eustatius and Saba	BES	Vital registration	Crude birth rate	National institution^26^	2015	3
Burkina Faso	BFA	Survey sample	ASFR	DHS^23^	2014	26
Bahamas	BHS	Vital registration	Birth count	National institution^27^	2013	18
Saint Barthelemy	BLM	Vital registration	Crude birth rate	National institution^28^	2013	1
Belize	BLZ	National estimate	ASFR	UNPD 2015^18^	2010–15	1
Bolivia	BOL	Survey sample	ASFR	DHS^23^	2008	18
Brazil	BRA	Vital registration	Birth count	National institution^29^	2015	5570
Barbados	BRB	National estimate	ASFR	UNPD 2015^18^	2010–15	1
Botswana	BWA	Vital registration	Birth count	National institution^30^	2014	15
Central African Republic	CAF	Survey sample	ASFR	DHS^23^	1994	11
Chile	CHL	Vital registration	ASFR	National institution^31^	2013	15
Cote D'ivoire	CIV	Survey sample	ASFR	DHS^23^	2012	21
Cameroon	CMR	Survey sample	ASFR	DHS^23^	2011	22
Congo, The Democratic Republic Of The	COD	Survey sample	ASFR	DHS^23^	2013	51
Congo	COG	Survey sample	ASFR	DHS^23^	2011	15
Colombia	COL	Survey sample	ASFR	DHS^23^	2010	64
Comoros	COM	Survey sample	ASFR	DHS^23^	2012	6
Cape Verde	CPV	National estimate	ASFR	UNPD 2015^18^	2010–15	1
Costa Rica	CRI	Vital registration	Birth count	National institution^32^	2015	7
Cuba	CUB	Sample survey	ASFR	MICS^33^	2014	16
Curacao	CUW	Vital registration	Birth count	National institution^34^	2013	1
Cayman Islands	CYM	Vital registration	Birth count	National institution^35^	2015	1
Djibouti	DJI	National estimate	ASFR	UNPD 2015^18^	2010–15	1
Dominica	DMA	Vital registration	ASFR	UNPD 2008^24^	2003	1
Dominican Republic	DOM	Survey sample	ASFR	DHS^23^	2013	18
Algeria	DZA	Survey sample	ASFR	MICS^33^	2012–13	14
Ecuador	ECU	Vital registration	ASFR	National institution^36^	2012	224
Egypt	EGY	Survey sample	ASFR	DHS^23^	2014	51
Eritrea	ERI	Survey sample	ASFR	National institution^37^	2010	6
Western Sahara	ESH	Survey sample	ASFR	DHS^23^	2003–04	4
Ethiopia	ETH	Survey sample	ASFR	DHS^23^	2011	21
Falkland Islands	FLK	Vital registration	Crude birth rate	National institution^38^	2008	1
Gabon	GAB	Survey sample	ASFR	DHS^23^	2012	19
Ghana	GHA	Survey sample	ASFR	DHS^23^	2014	20
Guinea	GIN	Survey sample	ASFR	DHS^23^	2012	15
Guadeloupe	GLP	National estimate	ASFR	UNPD 2015^18^	2010–15	1
Gambia	GMB	Survey sample	ASFR	DHS^23^	2013	14
Guinea-Bissau	GNB	Survey sample	ASFR	MICS^33^	2014	17
Equatorial Guinea	GNQ	National estimate	ASFR	UNPD 2015^18^	2010–15	1
Grenada	GRD	Vital registration	ASFR	UNPD 2015^18^	2010–15	1
Guatemala	GTM	Survey sample	ASFR	DHS^23^	2014–15	45
French Guiana	GUF	National estimate	ASFR	UNPD 2015^18^	2010–15	1
Guyana	GUY	Survey sample	ASFR	DHS^23^	2009	14
Honduras	HND	Survey sample	ASFR	DHS^23^	2011	38
Haiti	HTI	Survey sample	ASFR	DHS^23^	2012	21
Jamaica	JAM	Vital registration	Birth count	National institution^39^	2013	14
Kenya	KEN	Survey sample	ASFR	DHS^23^	2015	10
Saint Kitts and Nevis	KNA	Census	ASFR	UNPD 2008^24^	2001	1
Liberia	LBR	Survey sample	ASFR	DHS^23^	2013	30
Libyan Arab Jamahiriya	LBY	National estimate	ASFR	UNPD 2015^18^	2010–15	1
Saint Lucia	LCA	National estimate	ASFR	UNPD 2015^18^	2010–15	1
Lesotho	LSO	Survey sample	ASFR	DHS^23^	2014	20
Saint Martin	MAF	National estimate	Crude birth rate	World Bank^40^	2014	1
Morocco	MAR	Survey sample	ASFR	DHS^23^	2003–04	17
Madagascar	MDG	Survey sample	ASFR	DHS^23^	2008	42
Mexico	MEX	Vital registration	Birth count	National institution^41^	2015	32
Mali	MLI	Survey sample	ASFR	DHS^23^	2015	17
Mozambique	MOZ	Survey sample	ASFR	DHS^23^	2011	21
Mauritania	MRT	Survey sample	ASFR	MICS^33^	2011	23
Montserrat	MSR	Vital registration	ASFR	UNPD 2008^24^	2004	1
Martinique	MTQ	National estimate	ASFR	UNPD 2015^18^	2010–15	1
Mauritius	MUS	National estimate	ASFR	UNPD 2015^18^	2010–15	1
Malawi	MWI	Survey sample	ASFR	DHS^23^	2015–16	54
Mayotte	MYT	National estimate	ASFR	UNPD 2015^18^	2010–15	1
Namibia	NAM	Survey sample	ASFR	DHS^23^	2013	26
Niger	NER	Survey sample	ASFR	DHS^23^	2012	15
Nigeria	NGA	Survey sample	ASFR	DHS^23^	2015	72
Nicaragua	NIC	Survey sample	ASFR	DHS^23^	2001	34
Panama	PAN	Vital registration	Birth count	National institution^42^	2012	12
Peru	PER	Survey sample	ASFR	DHS^23^	2012	48
Puerto Rico	PRI	Vital registration	Birth count	National institution^43^	2009–10	78
Paraguay	PRY	Vital registration	Birth count	National institution^44^	2014	18
Reunion	REU	National estimate	ASFR	UNPD 2015^18^	2010–15	1
Rwanda	RWA	Survey sample	ASFR	DHS^23^	2014–15	60
Sudan	SDN	Survey sample	ASFR	MICS^33^	2014	36
Senegal	SEN	Survey sample	ASFR	DHS^23^	2014	28
Saint Helena	SHN	Vital registration	Crude birth rate	National institution^45^	2013	1
Sierra Leone	SLE	Survey sample	ASFR	DHS^23^	2013	27
El Salvador	SLV	Survey sample	ASFR	MICS^33^	2014	28
Somalia	SOM	National estimate	ASFR	UNPD 2015^18^	2010–15	1
South Sudan	SSD	Survey sample	ASFR	MICS^33^	2010	20
Sao Tome and Principe	STP	Survey sample	ASFR	DHS^23^	2008	8
Suriname	SUR	National estimate	ASFR	UNPD 2015^18^	2010–15	1
Swaziland	SWZ	Survey sample	ASFR	MICS^33^	2014	16
Sint Maarten	SXM	National estimate	Crude birth rate	World Bank^40^	2013	1
Seychelles	SYC	National estimate	ASFR	UNPD 2015^18^	2010–15	1
Turks and Caicos Islands	TCA	Vital registration	ASFR	UNPD 2008^24^	2005	1
Chad	TCD	Survey sample	ASFR	DHS^23^	2014	41
Togo	TGO	Survey sample	ASFR	DHS^23^	2013	11
Trinidad and Tobago	TTO	National estimate	ASFR	UNPD 2015^18^	2010–15	1
Tunisia	TUN	Survey sample	ASFR	MICS^33^	2011–12	18
Tanzania, United Republic Of	TZA	Survey sample	ASFR	DHS^23^	2012	59
Uganda	UGA	Survey sample	ASFR	DHS^23^	2014	19
Uruguay	URY	Vital registration	Birth count	National institution^46^	2015	19
Saint Vincent and The Grenadines	VCT	Vital registration	Birth count	National institution^47^	2013	5
Venezuela	VEN	Vital registration	Birth count	National institution^48^	2012	335
British Virgin Islands	VGB	Vital registration	ASFR	UNPD 2008^24^	2004	1
Virgin Islands, U.S.	VIR	National estimate	ASFR	UNPD 2015^18^	2010–15	1
South Africa	ZAF	Vital registration	Birth count	National institution^49^	2015	9
Zambia	ZMB	Survey sample	ASFR	DHS^23^	2013	20
Zimbabwe	ZWE	Survey sample	ASFR	DHS^23^	2010	19
ASFR= Age Specific Fertility Rate, DHS=Demographic and Health Survey, MICS=Multiple Indicator Cluster Survey, UNPD=United Nations Population Division.						

**Table 4:** Example of standardised country table containing the ASFR values for regions in Haiti with separate entries for urban and rural areas.

**ISO**	**Region**	**Rural/Urban**	**ASFR 15_19**	**ASFR 20_24**	**ASFR 25_29**	**ASFR 30_34**	**ASFR 35_39**	**ASFR 40_44**	**ASFR 45_49**	**Year**
HTI	WEST	URBAN	0.03	0.15	0.17	0.13	0.08	0.10	0.00	2012
HTI	NORTH	URBAN	0.05	0.10	0.12	0.14	0.07	0.03	0.01	2012
HTI	CENTRAL	URBAN	0.05	0.10	0.22	0.20	0.09	0.06	0.00	2012
HTI	SOUTH	URBAN	0.03	0.10	0.14	0.17	0.07	0.07	0.02	2012
HTI	NIPPES	URBAN	0.01	0.09	0.18	0.13	0.09	0.04	0.00	2012
HTI	WEST	RURAL	0.07	0.18	0.20	0.19	0.18	0.07	0.01	2012
HTI	NORTH	RURAL	0.08	0.17	0.21	0.16	0.13	0.07	0.02	2012

**Table 5:** Example of standardised country table containing the number of registered births per age group for regions in Venezuela.

**Region**	**ISO**	**b_15_19**	**b_20_24**	**b_25_29**	**b_30_34**	**b_35_39**	**b_40_44**	**b_45_49**	**year**
1	VEN	111	101	106	58	41	12	4	2012
2	VEN	113	135	109	80	55	24	7	2012
3	VEN	879	1026	788	422	194	58	13	2012
4	VEN	111	131	110	87	53	22	9	2012
5	VEN	150	152	129	106	62	30	6	2012
6	VEN	15	16	15	12	7	4	1	2012
7	VEN	20	33	21	17	9	6	4	2012
8	VEN	347	502	444	299	141	34	0	2012
9	VEN	197	193	137	78	30	6	0	2012

**Table 6:** Name and description of datasets available for Africa and Latin America and Caribbean countries.

**Name**	**Description**	**Resolution**	**Files Format**	**University of Southampton DOI**
Births in Latin America and the Caribbean	Estimated live births per grid cell for 2015 for LAC for 50 countries	3 arc seconds	GeoTIFF	10.5258/SOTON/WP00529
Births in Africa	Estimated live births per grid cell for 2015 for Africa for 58 countries	3 arc seconds	GeoTIFF	10.5258/SOTON/WP00528
Pregnancies in Latin America and the Caribbean	Estimated pregnancies per grid cell for 2015 for LAC for 50 countries	3 arc seconds	GeoTIFF	10.5258/SOTON/WP00527
Pregnancies in Africa	Estimated pregnancies per grid cell for 2015 for Africa for 58 countries	3 arc seconds	GeoTIFF	10.5258/SOTON/WP00526

## References

[d1] University of Southampton WorldPop. 2017 https://dx.doi.org/10.5258/SOTON/WP00004

[d2] University of Southampton WorldPop. 2017 https://dx.doi.org/10.5258/SOTON/WP00138

[d3] University of Southampton WorldPop. 2017 https://dx.doi.org/10.5258/SOTON/WP00530

[d4] University of Southampton WorldPop. 2017 https://dx.doi.org/10.5258/SOTON/WP00529

[d5] University of Southampton WorldPop. 2017 https://dx.doi.org/10.5258/SOTON/WP00528

[d6] University of Southampton WorldPop. 2017 https://dx.doi.org/10.5258/SOTON/WP00527

[d7] University of Southampton WorldPop. 2017 https://dx.doi.org/10.5258/SOTON/WP00526

