# Supplementary material for: Gridded birth and pregnancy datasets for Africa, Latin America and the Caribbean
Source: Sci Data. 2018 May 22;5:180090. doi: 10.1038/sdata.2018.90 (PMC5963337; doi:10.1038/sdata.2018.90)
Supplement: Supplementary Information [file sdata201890-s2.docx]

Supplementary Information

Table of Contents

Supplementary Figure 1 …………………………………………………………………………….…………………………….………2

Supplementary Figure 2 …………………………………………………………………………….…………………………….………3

Supplementary Table 1 …………………………………………………………………………….………….………………….………4


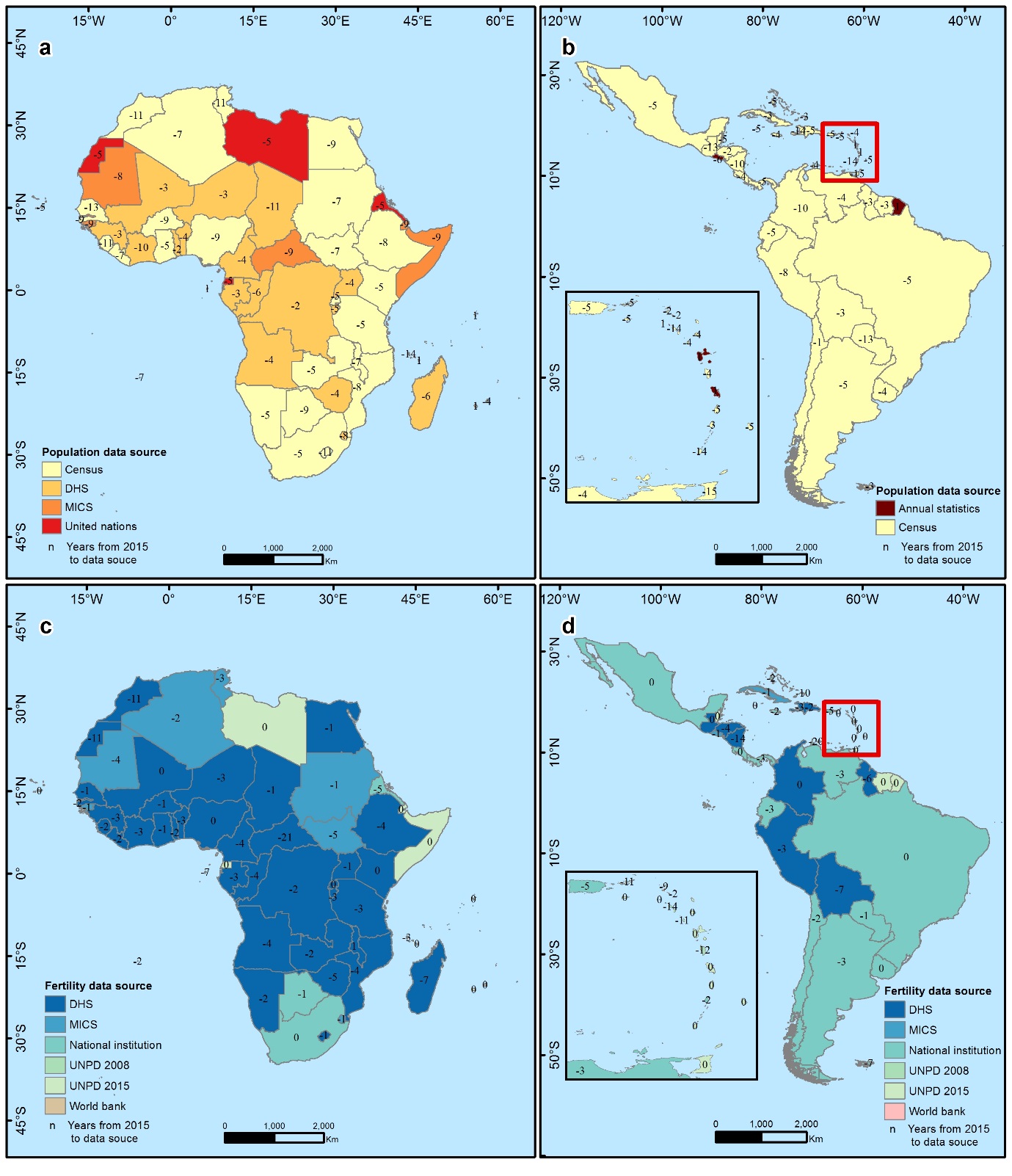


Supplementary Figure 1 Population and Fertility data source and date. Population (Age and Sex) data source and date for (a) Africa and (b) Latin America and the Caribbean. Fertility data source and date for (c) Africa and (d) Latin America and the Caribbean. DHS = Demographic and Health Survey, MICS = Multiple Indicator Cluster Survey, UNPD = United Nations Population Division. Further information in Table 3 of the manuscript.


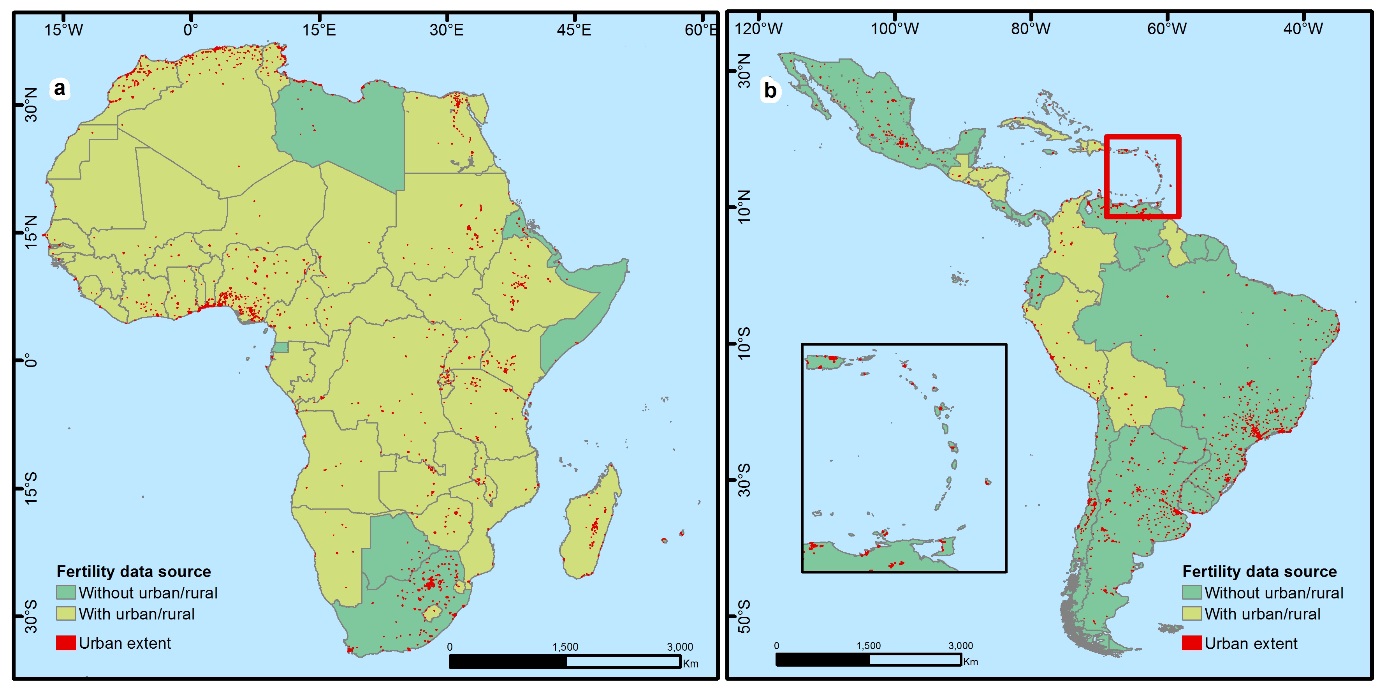


Supplementary Figure 2 Availability of fertility data disaggregated by urban and rural areas within each administrative unit for countries in (a) Africa and (b) South America and the Caribbean. Additionally, the urban extent used to allocate these values as described in the methodology in the manuscript is displayed (Methods Section).

| **ISO3** | **Country** | **Continent** | **Adjusted births UN** | **Unadjusted Births** |
| --- | --- | --- | --- | --- |
| AGO | Angola | Africa | 1068004 | 1179774 |
| BDI | Burundi | Africa | 456078 | 445970 |
| BEN | Benin | Africa | 373574 | 336672 |
| BFA | Burkina Faso | Africa | 687592 | 745813 |
| BWA | Botswana | Africa | 55095 | 41147 |
| CAF | Central African Republic | Africa | 160197 | 199964 |
| CIV | Cote D'ivoire | Africa | 801839 | 957845 |
| CMR | Cameroon | Africa | 823240 | 925089 |
| COD | Congo, The Democratic Republic Of The | Africa | 3049647 | 3370888 |
| COG | Congo | Africa | 162014 | 229341 |
| COM | Comoros | Africa | 25699 | 25787 |
| CPV | Cape Verde | Africa | 11022 | 10210 |
| DJI | Djibouti | Africa | 22089 | 19835 |
| DZA | Algeria | Africa | 949492 | 937356 |
| EGY | Egypt | Africa | 2477179 | 2729091 |
| ERI | Eritrea | Africa | 173790 | 211917 |
| ESH | Western Sahara | Africa | 10383 | 13058 |
| ETH | Ethiopia | Africa | 3107466 | 3746644 |
| GAB | Gabon | Africa | 50368 | 65105 |
| GHA | Ghana | Africa | 865439 | 1006827 |
| GIN | Guinea | Africa | 443774 | 422247 |
| GMB | Gambia | Africa | 78818 | 78216 |
| GNB | Guinea-Bissau | Africa | 65631 | 74064 |
| GNQ | Equatorial Guinea | Africa | 27953 | 28916 |
| KEN | Kenya | Africa | 1530325 | 1609075 |
| LBR | Liberia | Africa | 151237 | 191757 |
| LBY | Libyan Arab Jamahiriya | Africa | 136045 | 134912 |
| LSO | Lesotho | Africa | 59863 | 70693 |
| MAR | Morocco | Africa | 707019 | 573761 |
| MDG | Madagascar | Africa | 789163 | 717786 |
| MLI | Mali | Africa | 727913 | 664157 |
| MOZ | Mozambique | Africa | 1044771 | 1277705 |
| MRT | Mauritania | Africa | 130194 | 134241 |
| MUS | Mauritius | Africa | 14309 | 14789 |
| MWI | Malawi | Africa | 633164 | 614702 |
| MYT | Mayotte | Africa | 7115 | 6974 |
| NAM | Namibia | Africa | 70235 | 87237 |
| NER | Niger | Africa | 900825 | 806087 |
| NGA | Nigeria | Africa | 6884724 | 7054759 |
| REU | Reunion | Africa | 13276 | 12734 |
| RWA | Rwanda | Africa | 360627 | 373456 |
| SDN | Sudan | Africa | 1286713 | 1519952 |
| SEN | Senegal | Africa | 546622 | 604006 |
| SHN | Saint Helena | Africa | NA | 59 |
| SLE | Sierra Leone | Africa | 225869 | 259231 |
| SOM | Somalia | Africa | 447222 | 435649 |
| SSD | South Sudan | Africa | 417520 | 625154 |
| STP | Sao Tome and Principe | Africa | 6297 | 7851 |
| SWZ | Swaziland | Africa | 37501 | 38019 |
| SYC | Seychelles | Africa | 1705 | 1546 |
| TCD | Chad | Africa | 595715 | 610350 |
| TGO | Togo | Africa | 248528 | 246023 |
| TUN | Tunisia | Africa | 201348 | 198208 |
| TZA | Tanzania, United Republic Of | Africa | 1966418 | 2388619 |
| UGA | Uganda | Africa | 1576319 | 1530442 |
| ZAF | South Africa | Africa | 1115308 | 914911 |
| ZMB | Zambia | Africa | 611303 | 699566 |
| ZWE | Zimbabwe | Africa | 533373 | 477477 |
| ABW | Aruba | LAC | 1061 | 1211 |
| AIA | Anguilla | LAC | NA | 213 |
| ARG | Argentina | LAC | 754168 | 724025 |
| ATG | Antigua and Barbuda | LAC | 1479 | 1569 |
| BES | Bonaire, Saint Eustatius and Saba | LAC | NA | 295 |
| BHS | Bahamas | LAC | 5767 | 4734 |
| BLM | Saint Barthelemy | LAC | NA | 92 |
| BLZ | Belize | LAC | 7917 | 7934 |
| BOL | Bolivia | LAC | 251633 | 407549 |
| BRA | Brazil | LAC | 3073809 | 2913270 |
| BRB | Barbados | LAC | 3449 | 3641 |
| CHL | Chile | LAC | 235608 | 258438 |
| COL | Colombia | LAC | 764090 | 909555 |
| CRI | Costa Rica | LAC | 70768 | 71343 |
| CUB | Cuba | LAC | 119247 | 128875 |
| CUW | Curacao | LAC | 2027 | 1991 |
| CYM | Cayman Islands | LAC | NA | 744 |
| DMA | Dominica | LAC | NA | 1148 |
| DOM | Dominican Republic | LAC | 218370 | 240360 |
| ECU | Ecuador | LAC | 329648 | 215973 |
| FLK | Falkland Islands | LAC | NA | 26 |
| GLP | Guadeloupe | LAC | 6340 | 5526 |
| GRD | Grenada | LAC | 2048 | 1658 |
| GTM | Guatemala | LAC | 430372 | 408706 |
| GUF | French Guiana | LAC | 6556 | 6804 |
| GUY | Guyana | LAC | 14282 | 18262 |
| HND | Honduras | LAC | 169304 | 237506 |
| HTI | Haiti | LAC | 264088 | 326331 |
| JAM | Jamaica | LAC | 48705 | 39353 |
| KNA | Saint Kitts and Nevis | LAC | NA | 902 |
| LCA | Saint Lucia | LAC | 2810 | 2904 |
| MAF | Saint Martin | LAC | NA | 499 |
| MEX | Mexico | LAC | 2369241 | 1609165 |
| MSR | Montserrat | LAC | NA | 57 |
| MTQ | Martinique | LAC | 4637 | 4087 |
| NIC | Nicaragua | LAC | 124031 | 213156 |
| PAN | Panama | LAC | 74875 | 75304 |
| PER | Peru | LAC | 619628 | 806054 |
| PRI | Puerto Rico | LAC | 44696 | 42321 |
| PRY | Paraguay | LAC | 139146 | 66435 |
| SLV | El Salvador | LAC | 106693 | 129769 |
| SUR | Suriname | LAC | 9862 | 9935 |
| SXM | Sint Maarten | LAC | NA | 572 |
| TCA | Turks and Caicos Islands | LAC | NA | 547 |
| TTO | Trinidad and Tobago | LAC | 19805 | 20220 |
| URY | Uruguay | LAC | 49119 | 49277 |
| VCT | Saint Vincent and The Grenadines | LAC | 1797 | 1856 |
| VEN | Venezuela | LAC | 600817 | 610286 |
| VGB | British Virgin Islands | LAC | NA | 518 |
| VIR | Virgin Islands, U.S. | LAC | 1494 | 1488 |

Supplementary Table 1 Summary of adjusted births (UN) and unadjusted birth following methods described in the manuscript (Methods Section).
